# Mind–Body Therapy for Children with Attention-Deficit/Hyperactivity Disorder

**DOI:** 10.3390/children4050031

**Published:** 2017-04-25

**Authors:** Anne Herbert, Anna Esparham

**Affiliations:** 1 Gottlieb Memorial Hospital, Loyola University Health System, 701 North Ave, Melrose Park, IL 60160, USA; anne.herbert@luhs.org; 2 Division of Integrative Medicine, University of Kansas Medical Center, 3901 Rainbow Blvd, Mailstop 1017, Kansas City, KS 66160, USA

**Keywords:** attention-deficit/hyperactivity disorder (ADHD), mind–body, pediatric, children, mindfulness, meditation, yoga, integrative medicine, alternative, complementary

## Abstract

Attention-deficit/hyperactivity disorder (ADHD) is pervasive among the pediatric population and new treatments with minimal adverse effects are necessary to be studied. The purpose of this article is to review current research studying mind–body therapies for treatment of children diagnosed with ADHD. Literature was reviewed pertaining to the effectiveness of movement-based therapies and mindfulness/meditation-based therapies for ADHD. Many positive effects of yoga, Tai Chi, physical activity, and meditation may significantly improve symptoms of ADHD among children.

## 1. Introduction

Attention-deficit/hyperactivity disorder (ADHD) is a widespread chronic disorder affecting children’s well-being and success in life. The historical understanding of ADHD has changed over the years [1]. ADHD was defined in the Diagnostic and Statistical Manual of Mental Disorders (DSM)-III-R in 1987 as a disorder with a specific diagnostic checklist and three subtypes: primarily inattentive, primarily hyperactive, and combined. According to the American Psychiatric Association in the DSM-V, to be considered ADHD, a child must have symptoms before the age of 12, for at least six months, and affecting two domains of life. The prevalence of the three subtypes of ADHD are: primarily inattentive (20–30% of diagnosed population), primarily hyperactive-impulsive (less than 15%), and combined subtype (50–75%) [2]. The prevalence of ADHD in the US among children is estimated at 11% [3]. ADHD is very common among children and adolescents, consisting of about 50% of child psychiatric diagnoses [1]. 

## 2. Attention-Deficit/Hyperactivity Disorder

### 2.1. Etiology

A landmark theory about the primary dysfunction of ADHD was developed by Russel Barkley called the Hybrid Neuropsychological Model of Executive (Self-Regulatory) Function [4]. This theory explains ADHD as primarily a disorder of maladaptive behavioral inhibition and impairment of four executive functions. According to Barkley, behavioral inhibition has three functions: inhibiting a prepotent response, stopping an ongoing response, and controlling interference. Behavioral inhibition creates an opportunity for the executive functions to take place leading to a response. The four executive functions Barkley identified are (a) working memory, (b) self-regulation of affect, motivation and arousal, (c) internalization of speech, and (d) reconstitution. In individuals with ADHD, behavioral inhibition is dysfunctional, thereby impairing the four executive functions. In turn, motor response in the forms of motor control, fluency and syntax are affected because these actions are controlled directly by the four executive functions. Therefore, individuals with ADHD have a cascade of dysfunction, beginning with a deficit in behavioral inhibition, leading to an impairment of the four executive functions, resulting in altered motor responses [4]. Barkley’s theory must be considered in the present literature review regarding how mind–body therapy has the potential to affect executive functioning in ADHD. 

ADHD has been correlated with many different genetic and environmental influences. It has been identified that genetic factors, environment, brain structure, neural pathways, and neurotransmitter levels influence ADHD and its symptoms [2,5,6]. In-utero influences such as alcohol and tobacco exposure during pregnancy, low birth weight, toxemia, eclampsia, poor maternal health, maternal age, and certain complications during labor can predispose a child to ADHD. Furthermore, psychosocial adversity in a child’s life such as marital discord, low socioeconomic level, paternal criminality, maternal mental disorders, large family size, or foster care placement have been correlated with an increased risk for ADHD [5]. Further areas of study may be necessary to include the mechanism of action on how these predisposing risks affect executive function in children with ADHD.

ADHD has been identified as a *complex trait* disorder meaning that it is affected by many susceptibility genes. Each gene affects the risk of developing the disorder on a small scale (p. 51) [6]. Research on genetic associations with ADHD exhibit significant amounts of inconsistent results most likely due to its complexity. Twin and adoption studies show that genetic factors may account for over 70% of the of ADHD symptom variance. Mean heritability for ADHD has been shown to be 77% in twin studies [5]. An extensive meta-analysis by Gizer et al. reviewed the candidate genes associated with ADHD. Their analysis showed significant associations for several genes that influence neurotransmitter function including serotonin, dopamine and norepinephrine regulation. These were found to include *DAT1*, *DRD4*, *DRD5*, *5HTT*, *HTR1B*, and *SNAP25* [6]. One of the most extensively studied was the candidate gene *DAT1*, which codes for a carrier protein that allows for the reuptake of dopamine into the presynaptic neuron [6]. Studies also show genetic differences for the norepinephrine transporter (*NET*) that affects ADHD functioning [7,8]. Dysregulation of dopamine and norepinephrine is thought to play a role in ADHD symptoms [5,7]. The genetic variations associated with ADHD decrease norepinephrine and dopamine activity in the synapses. Stimulant medications, such as methylphenidate and amphetamine, may help overcome this by increasing the availability of norepinephrine and dopamine by inhibiting transporter functions [7]. 

A longitudinal study among children showed that children homozygous for the major A allele for *NET* were more likely to have a lifetime diagnosis of ADHD [7]. The major A allele may also be associated with higher ADHD symptoms scores. Sigurdardottir et al. found that individuals with the major A allele showed higher *NET* binding potential than controls. Higher *NET* availability was found to be associated with higher symptom scores on the Conners’ Adult ADHD Rating Scale: The Self-Report Screening Version (CAARS-S:SV) and Observer-Screen Version (CAARS-O:SV) [8]. Specific brain regions and neurotransmitters have been identified as playing a role in ADHD symptoms. Previous theories explain that the prefrontal cortex of the brain has the overall function of forming “cross-temporal structures of behavior that have a unifying purpose or goal” (p. 71) [4]. The prefrontal cortex is hypothesized to have a role in self-regulation of drive and motivational states that precede goal-directed actions. Because of this, the prefrontal cortex and its executive functions have an influence over motor control in the form of response, anticipatory setting to respond, or inhibition of response. Persons with damage to the prefrontal cortex present with similar symptoms to those with ADHD, having deficient inhibition and executive functioning [4]. Decreased volume of total cerebral brain matter in the frontal cortex, cerebellum and subcortical structures are also associated with ADHD. Hypofunction of anterior cingulate cortex may be associated with disinhibition in ADHD. Furthermore, frontosubcortical pathways that operate with catecholamines are affected in ADHD. Stimulant medications act on these pathways to increase inhibition of frontal cortical activity on subcortical regions. In addition, the corpus callosum and cerebellum play a role in cognitive functioning. Communication between the two regions may be degraded in ADHD [2]. 

Certain brain wave activity and patterns have been found among individuals with ADHD that set them apart from their counterparts. Electroencephalography (EEG) is used to analyze brain activity in individuals with ADHD. EEG studies can be analyzed both quantitatively and qualitatively. Certain studies investigate waveform amplitude, absolute and relative power, dominant and subordinate frequency analysis, wave percentage time, and coherence between regions. EEG provides a measure of the “background state” of the brain. Since the 1930s, EEG studies have identified that a subgroup of children with ADHD tend to have an increase in theta and delta slow wave activity, mostly in the frontal region [1]. During inattentive or unfocused states, the slow theta waves (3.5–8.0 Hz) dominate the prefrontal and frontal cortices, as well as other midline loci of the brain. During relaxed, wakeful states, alpha waves (9.0–11 Hz) take over these areas of the brain. Increasing awareness, planning, and purposeful actions cause the sensorimotor rhythm (12–15 Hz) to appear in the motor cortex [9]. Training the sensorimotor rhythm through neurofeedback has been hypothesized to improve inhibitory responses and control of attention in children with ADHD [10,11]. Focused attention or sustained mental effort causes beta-1 (13–21 Hz) and beta-2 activity (22–30 Hz) to be active in the prefrontal, frontal and central midline areas (p. 434) [9]. As the brain moves from a sleeping to attentive state, the wave frequency in central, midline and frontal regions increases and the amplitude decreases. Many individuals with ADHD have been shown to exhibit increased theta/beta wave ratios. Individuals with ADHD also exhibit differences in Event-Related Potential (ERP) tests. The ERP tests evaluate the brain’s electrical response to stimuli directly after the stimulus. It is conducted by presenting two types of stimuli, “Go” and “No-Go.” When presented with the “Go” stimulus, the participant must respond, while when the “No-Go” stimulus is presented, the participant is supposed to inhibit a response. According to the review by Monastra et al., participants with ADHD perform significantly poorer on “Go,” “No-Go” tests. Slow Cortical Potentials (SCPs) are event-related current shifts in the brain that originate from the upper cortical layer and last 300 msec to a few seconds. Negative electrical shifts have been linked to reduced activation of regions related to orientation and attention, which is of interest to ADHD research. SCP biofeedback training is currently being studied where participants in this treatment receive “feedback” related to their regulation of positive or negative current shifts. Reduced ADHD symptoms were reported in two studies of SCP biofeedback training, but there is not enough controlled research to be able to consider it as an effective treatment at this time [9]. However, it is not yet universal that EEG-neurofeedback can diagnose ADHD based on brainwaves as it may only differentiate a relatively small group of these children.

Lazzaro et al. investigated brain wave activity of adolescents with ADHD compared to age- and sex-matched counterparts [12]. The study recruited 54 male adolescents 11–17 years of age, 34 were unmedicated, and the rest were taken off of medication two weeks prior to the study. They were age-matched with 54 male adolescents without ADHD as a control group. The participants underwent a baseline resting, open-eye EEG and then the experimental EEG where they were asked to focus their sight on a black dot 60 cm away to limit eye movement. Brain activity was then measured for two minutes. There was significantly increased theta and alpha-1 activity across anterior, midline, posterior, left and right hemisphere brain regions among ADHD participants. Delta and alpha-2 activity was not significantly increased among ADHD participants compared to control [12]. In summary, the majority of individuals with ADHD presented with excess theta activity and decreased beta in the frontal and central midline regions, also known as cortical hypoarousal. However, there is a minority of individuals with ADHD who exhibit cortical hyperarousal or no changes in quantitative electroencephalography (QEEG) compared to healthy controls. As far as diagnostic value of QEEG, the reported sensitivity and specificity of the theta/beta power ratio has been shown to be comparably accurate in differentiating ADHD from healthy counterparts when compared with behavioral rating scale measures [9]. 

Heart rate variability (HRV) may be a useful marker for therapeutic changes in ADHD and illustrates the relationship between ADHD and the autonomic nervous system. Abnormal catecholaminergic function has been linked to ADHD, resulting in “underarousal of the sympathetic system in children with ADHD” (p. 1366) [13]. Stimulants increase the dopamine and norepinephrine activity. In turn, studies have shown increases in blood pressure and heart rate due to these medications. One study consisting of 37 children with ADHD, ages 6–12 years, showed a significant change in HRV from baseline to endpoint. Prior to the study, children were either medication naive or taken off medication for at least two months. Square root of the mean squared difference of successive normal-to-normal intervals (RMSSD) and high frequency (HF) were calculated as indicators of parasympathetic vagal tone. Both of these measures were significantly decreased after introduction of methylphenidate treatment from baseline to endpoint. Average heart rate significantly increased from baseline to endpoint. These results may indicate that the children with ADHD may exhibit decreased parasympathetic activity as indicated by increased HRV following pharmacological intervention [13]. Another study by Rukmani et al. exhibited similar results, finding that medication-naive children with ADHD had reduced overall heart rate variability compared to age and gender-matched controls. They hypothesized that these results indicate catecholamine dysregulation and parasympathetic dominance in ADHD [14]. However, this needs to be further studied due to the heterogeneity of ADHD, as catecholamine and the autonomic nervous system imbalance may be different in subgroups of individuals with ADHD. Mind–body therapies may offer an effect on functioning of the autonomic nervous system. Future research should investigate effects of mind–body therapy on measures of heart rate variability in order to compare its effects to that of pharmacological treatment. 

### 2.2. Current Treatments

ADHD presents with a complex etiology and has multiple treatment options. Stimulant medication is the most often used medication for the disorder in younger children, and more recently, non-stimulant medications are used for children and adolescent populations. Other treatments include behavior modification, which includes therapy from clinicians, family or parent training and therapy, and school-based interventions in the classroom [15].

A recent study of 152 children with ADHD revealed that initial treatment behavior modification was less costly than initial treatment with a low dose of stimulant medication. This experimental study found that, on average, children who underwent successful behavior modification training had a cost of $392 versus $1448 for pharmacological treatment over a 10-month school year [16]. In addition, the Multimodal Treatment Study demonstrated that medication did have any long-term benefits in reducing ADHD symptom severity [17]. Adverse effects that are most common of stimulant medications are appetite loss, abdominal pain, headaches, and sleep disturbance [15]. Common effects of non-stimulant medications, such as atomoxetine, include initial somnolence, gastrointestinal disturbances, and decreased appetite. Other non-stimulant medications, such as clonidine or guanfacine, may cause somnolence and dry mouth [15]. 

Behavioral interventions and pharmaceutical regimens have been shown to be effective at reducing ADHD symptoms significantly, especially when used in combination [18]. It deserves to be explored if the integration of mind–body therapy, with these already accepted treatments would further decrease ADHD symptoms and improve functioning. Mind–body therapies offer a wide range of effects on psychosocial, emotional, and neurobiological functioning, making it a promising addition to current therapy regimens.

## 3. Mind–Body Therapies

### 3.1. Current Treatments

Integrative medicine offers an alternative or addition to pharmacological or cognitive-behavioral therapies for treatment of ADHD. There are limited well-designed studies of integrative therapies, making it difficult to generalize results. Current therapies to improve ADHD symptoms identified through evidence-based medicine include diet, nutrient supplementation, gut health, environmental health, and neurofeedback [19].

Mind–body therapy includes but is not limited to mindfulness, biofeedback, deep breathing, guided imagery, progressive relaxation, hypnotherapy, and yoga [20]. A reason for incorporating more integrative therapies is motivated by consumer interest and the need to improve treatment outcomes. Mind–body medicine encompasses various therapies that are non-invasive techniques with the aim of “harnessing positive thought and emotion for the purpose of enhancing health” (p. 102) [20]. The practice of mindfulness and meditation is a conscious exercise that builds attention control and inhibitory skills [21]. 

### 3.2. Yoga

One field of mind–body medicine is the practice of yoga. Yoga is an ancient physical practice, derived from Sanskrit word “yuj” meaning “to yoke,” and refers to uniting the body, mind and spirit. Yoga teaches individuals to master certain poses and breathing techniques which may promote self-control, attention, awareness and adaptive skills. Yoga has been used as a form of exercise and meditation. It has been currently studied as therapy for the treatment of stress, chronic pain, asthma, irritable bowel syndrome, and ADHD [20].

Yoga has various components to unite the body, mind and spirit. It is comprised of the asanas (physical postures), pranayamas (breathing techniques), and dharana/dhyana (meditation practices) [22]. The goal of these components is to connect the breathing, thoughts, emotions, and body into awareness of the present moment. The conditions affected by yoga explored by Rosen et al. included emotional, mental and behavioral health. The studies reviewed showed significant decreases among children practicing yoga in a variety of measures such as: negative stress response behaviors, total mood disturbance, negative affect, anger, resilience, and fatigue/inertia [22]. There are few studies that may suggest that yoga influences HRV and autonomic function, but more rigorous studies are needed to report any firm conclusions [23]. 

One study by Hariprasad et al. investigated the effects of yoga as a complementary therapy for children with ADHD who were admitted in a child psychiatry unit [24]. The sample was made of nine children, ages 5–16 years. Eight of the nine children were on medications. The children underwent eight yoga sessions over the course of their inpatient stay. The children were rated on Conners’ abbreviated rating scale (CARS), ADHD-rating scale IV (ADHD-RS IV), and clinical global impression (CGI)-Severity. They were rated at the beginning of the study, discharge, and at one month, two months and three months following the study. It was found that CARS, and ADHD-RS IV, and CGI-S were significantly improved among the patients upon discharge. Confounding variables are that this study lacked a control group and the group represents a more severely impaired population of ADHD individuals as evidenced by their need for inpatient treatment. Additionally, the children were taking medication and influenced by other inpatient interventions besides yoga. While yoga may offer some benefit as a complementary therapy in an inpatient setting, this study lacks controls and a large enough sample needed to recommend yoga as an add-on intervention [24].

Sahaja yoga practice as family therapy may have a positive effect on ADHD symptoms and could possibly reduce dosage of medication in some cases. Another study investigated effects of Sahaja yoga meditation as a family treatment for children with ADHD [25]. There were 48 participants in the study, 31 receiving medication, 14 not on medication, and 3 unknown. The participants went through a six-week program of 90-minute clinic sessions twice a week and regular meditation at home. Effects of the yoga were measured by pre- and post-assessment of child self-ratings and parent’s rating of ADHD symptoms, self-esteem and child–parent relationship quality. The results of the study indicated that parents reported significant improvements in ADHD symptoms, with an average decrease calculated at 35%. The researchers also compared scores of the six non-medicated versus the medicated children and found that there were no significant differences in symptoms, indicating that the changes were likely not due to pharmacologic treatment. In addition, 11 of the 20 children who were medicated could reduce their dose of medication throughout the yoga program. In addition to improvement in symptoms, parents also reported improved self-esteem and relationships, feeling less stressed and had a better ability to handle their children’s behavior. Children self-reported better sleep patterns, less anxiety, more ability to focus at school, and less conflicts. However, this study relies on unblended ratings of parents and lacked a control group, which makes it difficult to infer that Sahaja yoga may be an effective adjunctive therapy for ADHD. Sahaja yoga has also been reported to increase gray volume matter associated with sustained attention, self-control, compassion, and interoceptive perception in older adults [26]. Additionally, the yoga as a form of stress reduction for parents of children with ADHD is a valid topic to be explored by future research.

Yoga may also help increase time on task for students with ADHD in the educational setting. One study investigated the effect of a yoga practice intervention on children’s time on task in school [27]. The participants were 10 children, ages 6–10, with attention problems. The children completed three weeks of two sessions per week for 30 min in each session. The time to task was measured by observation during morning class work in school. The observers used the Behavioral Observation Form where time on task was defined as the percentage of intervals when students had eye contact with the teacher or assigned task and performed assignments. Cohen’s effect size was calculated, with small effect being 0.20 or greater, moderate 0.50 or greater and, large effect 0.80 or greater. Effect size for each grade was calculated by taking the difference between the mean of the baseline and intervention phases divided by the standard deviation of the baseline phase. The effect sizes regarding behavioral observation scores were found to be from 1.51 to 2.72 for the three grade groups, showing a large effect. At follow-up, the effect sizes were decreased, but still showing a moderate to great effect at 0.77 and 1.95. Observation as a measurement of student behavior has both strengths and limitations. A limitation is that observation may include rater bias, especially when the observer is not blinded to the purpose of the study as was the case in the present study. On the other hand, teacher-ratings are often used in ADHD research such as the Conner’s parent/teacher rating scale. Another positive result of this study was that children reported enjoying the yoga videos and that video format is an inexpensive and easy method of presenting the therapy. Yoga may have the potential to increase time on task in class and be an enjoyable and cost-effective treatment for children with attention problems [27]. 

There are few experimental studies exploring the effects yoga on attention mechanisms. One study investigated how 20 yoga sessions for boys with ADHD would help attention versus a control of cooperative game activities [28]. The experimental group consisted of 11 participants and control of eight boys. The two groups were assessed pre-and post-intervention using the Conners’ Parent and Teacher Rating scale and scores were compared by one-way ANOVA. Qualitative data was also taken from parents. The scores in five subscales of the measurement were improved for the yoga group post-intervention. Parents also reported that the relaxation and breathing techniques were beneficial when their children were restless, needed sleep, and affected their behavior in the immediate period after class. The participants in both groups were receiving medication throughout the study. This experiment shows that yoga can be an effective complementary therapy for children with ADHD that are receiving medication [28]. The breathing and relaxation techniques that are learned through yoga practice may be used by children to focus attention and decrease hyperactivity.

In reference to the theory of executive dysfunction being the hallmark of ADHD, yoga practice must be evaluated in regards of its effect on these functions. Chou and Huang found that an eight-week yoga program significantly improved sustained attention and discrimination function in children with ADHD compared with a control group [29]. The researchers created two groups of children 8–12 years old from the same suburban area: yoga (*n* = 24) and control (*n* = 25). The two groups were matched, so there were no significant differences in extraneous variables. They measured children at baseline and after intervention on the Visual Pursuit Test and Determination Test. These types of measurements may limit the application of this study because these two tests have not been used among ADHD populations. However, the Visual Pursuit Test is used in psychological diagnostics for selective and sustained attention and was used because attention deficits are symptoms of ADHD. Additionally, the Determination Test assesses for reaction speed, attention deficits, and reactive stress tolerance in the presence of external sensory stimuli. Findings revealed that the group who underwent yoga intervention had significantly better reaction time and response accuracy at the two measurements [29]. 

Another recent study investigated yoga and physical activity on measures of executive functioning [30]. They examined 30 female undergraduate students’ scores on the Flanker task, a measure relating to inhibitory control, and the N-back task, a measure relating to working memory, under three different conditions: baseline, aerobic exercise and yoga. It was found that response accuracy to the Flanker task was significantly increased under the yoga condition compared to aerobic exercise. The researchers believe that the positive effects exhibited under the yoga condition on the two measures may be due to improvements in mood and its focus on body awareness and breathing. By these mechanisms, it is hypothesized that yoga would have positive effects on attentional ability [30].

### 3.3. Tai Chi

Tai Chi is an ancient Chinese martial art consisting of slow movements coordinated with balancing body weight and breathing deeply. There may be potential benefit for improving ADHD symptoms of anxiety and hyperactivity with regular Tai Chi practice. A sample of 13 adolescents with ADHD was studied to find the effect of a five-week Tai Chi intervention on symptoms according to the Conners’ Scale rated by teachers. The participants were rated at baseline, at the end of the five weeks and two weeks after intervention. During the five weeks, participants completed two 30-minute sessions per week. It was found that the adolescents exhibited significantly decreased anxiety, decreased daydreaming, decreased inappropriate emotions, decreased hyperactivity, and increased conduct after the Tai Chi intervention. The scores persisted to be significantly affected even at the two-week post-intervention post-test [31]. Researchers hypothesized that these effects may be due to lower levels of stress and cortisol release, which has been shown as an effect of Tai Chi in previous research studies [32]. According to the theory that executive functions are impaired in ADHD, Tai Chi may help improve executive functioning because it requires a combination of focused attention on movement and breath as guided by the teacher. This study shows that Tai Chi may have some beneficial effects on adolescents with ADHD as an adjunctive therapy [31]. 

### 3.4. Physical Activity

In human studies, physical activity and fitness has shown to affect brain structure and increase brain activity that is associated with conflict and attentional control. Though ADHD is a disorder with a complex etiology, there are a few factors contributing to the likelihood of ADHD that may be affected by physical activity. Exercise has also been shown to improve dopamine function, known to be dysregulated in ADHD [33]. Brain derived neurotrophic factor (BDNF) has also been found to play a role in ADHD symptoms through recent research [34]. This factor helps development of neurons in the dopaminergic pathways in the brain, which may be of importance to impulse control and attention. According to animal studies, BDNF expression is increased with stimulant medications [35]. The prefrontal and frontal regions contribute to regulation of catecholaminergic pathways involved in ADHD. According to Madras et al., there may be a decreased ability for people with ADHD to reload these catecholamines effectively [36]. Physical activity may affect these same neurological factors and brain regions. It has been shown that physical activity increases cerebral blood flow, thereby potentially helping where ADHD exhibits a deficit in blood flow to the prefrontal and frontal regions. Hunter et al. demonstrated that physical activity may increase the amount of dopamine and norepinephrine in synaptic clefts which activate catecholaminergic pathways [37]. High impact running significantly increased measurements of dopamine, epinephrine and BDNF activity. Additionally, these increases were correlated with significant improvements in memory and learning ability tasks among the study participants [37].

A recent meta-analysis demonstrated that physical activity increases executive function and cognitive functioning with an effect size of 0.535 [38]. In another meta-analysis, pre-adolescent children were found to have increased executive function after one aerobic exercise session (effect size 0.540) [39]. One study investigating karate and its impact on motor and cognitive development showed that this particular martial arts form resulted in improved attention through the Tower of London test (effect size 0.88, *p* < 0.05) [40]. Martial arts teach children to practice self-control, concentration and meditation that may ultimately result in improvements of executive function. A study by Medina et al. showed that boys with ADHD had significantly improved attention after high-intensity treadmill exercise lasting for 30 min with or without medication [33]. The impact on cognitive function and academic performance needs to be further studied, as a recent systematic review found that the evidence for the effect of exercise on ADHD is equivocal and limited in quantity and quality [41]. While the preliminary evidence supports physical activity as an adjunctive treatment for ADHD, the data is insufficient to support it as a stand-alone treatment. A literature review by Gapin et al. suggests that future research should investigate physical activity as a stand-alone therapy for individuals that prefer alternative treatments as this topic has not been extensively tested in research [42]. 

## 4. Mindfulness-Based Therapies

Mindfulness and meditation have been found to “change brain activation patterns, contribute to enhanced mood, reduce anxiety, improve stress management, reduce pain and enhance immune function” [43] (p. 510). These effects imply that mind–body therapies could be well-suited for symptoms of ADHD in children. Mindfulness is “defined as a process of bringing one’s complete attention to the present experience on a moment-to-moment basis” (p. 64) [44]. In other descriptions, it has been known as bringing awareness to the present experience without a judgmental attitude, but rather attentive and curious [45]. Mindfulness was developed from ancient cultural meditation practices and has now been adopted by Western psychology and medicine as treatment for stress reduction therapy [44]. Additionally, mindfulness therapy has been found to be satisfying and without unwanted side effects for persons with ADHD [45,46]. Meditation has been defined as a mental training technique that can enhance an altered state of consciousness, resembling its similarity to mindfulness [47]. Mindfulness and meditation have also been found to increase connectivity amongst brain regions associated with executive function [48]. 

### Meditation/Mindfulness

The effect of an eight-week mindfulness training program for adults and adolescents with ADHD was considered [46]. The group consisted of 24 adults and eight adolescents who completed the intervention of eight weeks with 2.5 hours of training per day and additional “homework” meditation of 5 to 15 min. Measurements for pre- and post-intervention included the ADHD Rating Scale IV and SNAP-IV scale (for ADHD symptoms), the Attention Network Test (ANT) (for alerting, orienting and conflict attention), and the Stroop task (for attentional conflict), the Trail Making Test (for set-shifting and inhibition), the Digit Span (for working memory) in addition to other measurements. Substantial effects of the meditation were exhibited. In addition, 78% of participants reported a significant reduction in their ADHD symptoms with 30% reporting at least a 30% reduction. Significant improvement in attentional conflict and set-shifting was also found from pre- to post-intervention. Participant satisfaction was rated at a 9/10 on the visual analog scale and no adverse side effects were reported from the therapy. This study supports the hypothesis that mindfulness training may reduce self-reported ADHD symptoms and improved performance on certain attentional and cognitive tests [46]. 

This study by Crescentini et al. examined the effects of mindfulness training on 16 healthy primary school children ages seven to eight years old. The training consisted of three sessions per week for eight weeks [45]. A pre-test and post-test were taken by the main teacher of the students measuring behavior, social, emotion, and attention regulation skills. The children also self-reported mood and depression symptoms. There was a positive effect of the intervention on reduction of attention and internalizing issues. Children did not report any change in depressive symptoms after the intervention. This study shows support that mindfulness training has beneficial effects on attention and ADHD symptoms [45]. Mindfulness training can be incorporated into classroom activities or at home easily. Unlike medications, there are no harmful side effects and the training can be virtually at no cost to parents or teachers if they are educated on how to carry out the sessions for children 

The effects of meditation on attention were studied by Jha et al. [44]. Two experimental conditions were created to study mindfulness and its effect on attention. Three realms of attention were studied: alerting, orienting and conflict-monitoring. These areas of attention were measured by scores on the Attention Network Test (ANS) at Time 1 before treatment and Time 2 after treatment. Group 1 underwent an eight-week long, three-hour daily course. Group 2 underwent an intensive retreat for one month where they completed 10–12 h of mindfulness practice. Group 3 was a control group of persons with no previous mindfulness experience. Group 1 also had no previous mindfulness experience, while Group 2 had previous mindfulness practice and therefore could be compared as “experienced” in mindfulness. Results showed that participants in Group 1 had significantly improved scores of orienting-type attention and endogenous orient attention in comparison with the other two groups. Group 2 exhibited improved scores in exogenous stimulus detection when compared to the other two groups [44].

Another study by Van de Weijer-Bergsma et al. incorporated parents and tutors into the mindfulness intervention [49]. Ten adolescents, 19 parents and seven academic tutors underwent mindfulness training for eight weeks for 1.5 h sessions. The training consisted of sitting meditation, body scan, and breathing space exercises along with interventions to improve awareness of one’s distractability, impulsivity, and hyperactivity. Measurements were completed before the training as a pretest, at the end of the eight-week training and then again after 16 weeks. Measures consisted of scales measuring behavior problems, executive functioning, mindful awareness, parenting stress, parenting style, fatigue, happiness, and a computerized test of attention. Statistical analysis was carried out from pre-test to post-test to follow-up by paired *t*-tests with significant effects at *p* < 0.10 in a two-tailed *t*-test. At eight weeks, it was found that the adolescents had improvements in attention problems, executive functioning and performance on the computerized attention test [49]. The significance of their findings should be interpreted with caution, as the significance level is more liberal at *p* < 0.10. Further study should explore effects on mindfulness, perhaps with the same measurements but with larger sample sizes and interpreted with more strict significance scoring.

Studies show that meditative practice causes measurable changes within brain-wave activity. One experiment by Lagopoulos et al. investigated the effects of nondirective meditation on theta and alpha activity in the brain as recorded by EEG [50]. Eighteen participants who engaged in regular meditation for many years were recruited for the study. The experiment compared the theta and alpha activity between 20 minutes of non-directed meditation to non-meditative relaxation among the participants. There was a significant increase in alpha and theta activity among all three brain regions during meditation when compared to the relaxation session. Delta activity was significantly greater in the temporal central region during meditation as compared to relaxation. There was significantly greater alpha activity in the posterior region when compared to the frontal region, whereas theta activity was greater in the frontal and temporal-central regions compared to the posterior [50]. Meditation’s effects on brainwaves may be an important area of study to determine what brainwave changes occur in children with ADHD practicing mind–body therapies, such as meditation.

An important factor to consider in the discussion of mindfulness or meditation for ADHD treatment is the type of meditation being used. Certain forms of mindfulness lead participants to greater awareness or attention on breathing or emotions, while others encourage free, relaxed mental states of “inattention.” For example, one form of meditation, known as Yoga Nidra, is characterized by leading participants to “lose executive control” and has been shown to cause decreased blood flow to areas of the brain that regulate executive functions [51]. This study showed increased theta activity, which supports other meditation studies on brain wave activity. Because persons with ADHD already exhibit increased theta activity, it is counterintuitive that meditation could exhibit a beneficial effect on symptoms by causing a potential further increase. One potential mechanism by which meditation might affect ADHD symptoms is through the regulation of dopamine. Kjaer et al. found that dopamine release was significantly increased in the ventral striatum during Yoga Nidra meditation practice [51]. Through a C-raclopride Positron Emission Tomography (PET) scan, they found that there was as great as a 65% increase in dopamine among their participants [51]. Since medications for ADHD primarily target dopamine and norepinephrine transporters to increase synaptic levels, there may be a possibility that meditation could exhibit similar effects to these medications by the same means [7,51].

## 5. Conclusions

Potential benefits and mechanism of action of mind–body therapies have been evidenced through research and continue to be explored. Compared to pharmacological treatment, mind–body therapies have little to no unwanted side effects. There is little cost compared to clinical therapy since the only cost is for training or sessions that are typically conducted in groups. Activities such as yoga or Tai Chi can be practiced at home or school. Families and teachers can access videos online or purchase videos from reliable sources as resources to guide the therapy. 

Mindfulness and meditation can be done anywhere and at any time. Furthermore, mindfulness is a learned skill. It is counterintuitive that meditation has positive effects on ADHD symptoms because theta to beta ratios are typically increased in ADHD, and meditation further increases theta wave activity [48]. Meditation and mindfulness may improve symptoms not because of the quantity of brainwave activity, but because of the learned skill to control attention and focus to a specific purpose or action (i.e., the breath). Further study of whether persons with ADHD can perform meditation and mindfulness more effectively because of their naturally increased theta activity is worth exploration. How mind–body therapies affect neuroanatomical and neurotransmitter function may also support its therapeutic use. 

Mind–body training for parents has an added benefit to children’s ADHD symptoms. Parents who practice mindfulness with parenting techniques report better outcomes in ADHD symptoms of their children [48]. It is probable that the effects of the studies involving children and parents could be not only due to the intervention itself, but the lasting actions of the child–parent interaction at home following the mind–body sessions. A parent who learns mindfulness through yoga or meditation may have improved methods of disciplining and responding to a child’s behavior, and, in turn, the child may learn from the parent how to change their behavior in a positive way. Furthermore, having parents involved in the same treatment as their children helps continuation of mindful practice at home rather than limiting training to individual sessions.

Limitations of current research are primarily due to small sample sizes and lack of control groups. More research needs to be done with larger sample sizes and more controlled settings. Many of the studies reviewed also gathered data from subjective self-report or parent/teacher surveys. More objective data should be measured alongside subjective data in future research. 
